# Nutritional Status and Educational Performance of School-Aged Children in Lalibela Town Primary Schools, Northern Ethiopia

**DOI:** 10.1155/2020/5956732

**Published:** 2020-04-09

**Authors:** Muluken Ayalew, Alemayehu Bayray, Abate Bekele, Simegnew Handebo

**Affiliations:** ^1^Lalibela Hospital, Lalibela, Ethiopia; ^2^School of Public Health, College of Health Sciences, Mekelle University, Mekelle, Ethiopia; ^3^Department of Health Education and Behavioral Sciences, College of Medicine and Health Sciences, Institute of Public Health, Gondar University, Gondar, Ethiopia

## Abstract

**Background:**

Every student has the potential to do well in school. Failing to provide good nutrition puts them at risk for missing out on meeting that potential and leads to long-term irreversible damage to cognitive development. However, taking action today to provide healthier choices in schools can help set students up for a successful future full of possibilities. So, this study is aimed at assessing the effect of nutritional status on academic performance of school-aged children.

**Methods:**

A school-based cross-sectional study was conducted from Feb 05- March 30, 2017 in Lalibela town. A total of 505 primary school students were included in the study. The child anthropometric measures were calculated using AnthroPlus software version 1.0.4 WHO 2007 standards. Bivariable and multivariable ordinal logistic regression were fitted. The proportional odds ratio (POR) with a 95% CI and *P* value < 0.05 were considered statistically significant.

**Results:**

The overall prevalence of stunting, underweight, and thinness was 29.5%, 35.7%, and 29.5%, respectively. In this study, 14.7% of the study participants had low academic achievement in the semester. The multivariable analysis showed that rural residence (POR = 0.39; 95% CI: (0.21, 0.75**))**, not studying regularly (POR = 0.49; 95% CI: (0.29, 0.82)), severe underweight (POR = 0.25; 95% CI: (0.09, 0.71)), and severe stunting (POR = 0.03; 95% CI: (0.01, 0.37)) were associated with decreased odds of high average semester score achievement of school-aged children. Additionally, higher maternal educational status (POR = 2.12; 95% CI: (1.10, 4.07)) and family income from 550 to 2999 ETB (POR = 1.71; 95% CI: (1.04, 2.81)) were associated with increased odds of high semester average score achievement.

**Conclusion:**

Nearly one-third of school-aged children in Lalibela town were stunted, thin, and underweight. Rural place of residence, not studying regularly study, underweight, and stunting were associated with decreased academic achievement. While, family monthly income from 550 to 2999 ETB and higher maternal education status were associated with increased academic achievement.

## 1. Background

Globally, the magnitude and severity of malnutrition are still a public health problem. Malnourishment accounts for 2.6 million of deaths per year and more than 450 million children's failure in somatic and mental development [1]. Child undernutrition is a challenge of global health, especially in low- and middle-income countries [2]. Current evidences on children in Africa, Asia, and Latin America reported that the mortality of stunted and underweight children was greater than three times that of well-nourished children [3].

Anthropometric indices are commonly used to assess nutritional status, health, and development of children as well as the whole population [4, 5]. Nutritional status depends on dietary intake of food, nutrients, diseases and overall health status, and health care practices, which indirectly affects the educational performances [6]. Academic performance, intellectual development, and school attendance of the children and adolescents were adversely affected by undernutrition. On the other hand, good nutrition was associated with progress of perceptive and behavioral talents and improved school attendance [7, 8].

Empirical evidences witnessed the influence of nutritional status on academic achievement of the students. For example, in Indonesia, the cognitive development of preschool children was significantly affected by stunting, underweight, and severe wasting [9]. Additionally, low level of educational performance (marks < 40%) in Tamil (a language in Nuwara Eliya Educational Zone), mathematics, and overall subject average was significantly associated with underweight [10]. On the other hand, stunting is associated with decreased school-age enrolment and reduced achievement equivalent to three years of schooling as well as productivity deficit of greater than 20% [8, 11].

Though there are different issues raised for poor educational performances, 16% of the total repetition rate in primary education is highly associated with stunting in Ethiopia [12]. The participation rate of primary education is less than 22% of the relevant age cohort [13]. Similarly, realities in the country indicates that low educational performance rate of children with stunting is 15.1% and 11.2% with nonstunted children [14]. So, undernutrition was pointed to be associated with low educational achievements in primary education [15]. Higher risk of grade repetition and dropout of schools among age category of 6-18 years is still because of stunting [12].

Although there are several reports of malnutrition in Ethiopia, the relationship of nutritional status and educational performance of school-aged children was not yet explored in different areas of the country. Thus, this study was aimed at assessing nutritional status and academic achievements of primary school children in Lalibela, Northern Ethiopia.

## 2. Methods

### 2.1. Study Setting and Design

A school-based cross-sectional study was carried out from February 5 to March 30, 2017 in Lalibela town. Lalibela town is located in North Wollo Administrative Zone, which is 701 km from Addis Ababa, the capital of Ethiopia. According to the town health bureau population profile, the total population of the town is estimated to be 32,668. One district hospital and a health center provide health service in the town. With regard to education, one College, one preparatory school, one high school, nine primary schools, and two kindergarten schools are available in the town. There are about 2,992 total children in primary schools in the town [16].

### 2.2. Study Population

All school-aged children in Lalibela primary schools and those who lived in the town for at least six months were recruited in the study. The sample size was computed using Epi Info version 7, by considering the following assumptions: proportion of low academic performance in unexposed 34.9% [10], power = 80%, two − sided CI = 95%, ratio (unexposed : exposed) = 1, and OR = 1.667. By adding 10% nonresponse rate, the final sample size was 542. Four public schools were selected randomly from nine primary schools in Lalibela town. The sample size was distributed proportionally to the selected schools. Then, simple random sampling method was employed to select each study participant.

### 2.3. Data Collection Procedures

Structured, pretested, and interviewer-administered questionnaires were used for data collection. First, the questionnaire was prepared in English and translated into the local language, Amharic, and then translated back to English to check the consistency. Mothers or caregivers were interviewed on socioeconomic and other demographic data. Anthropometric data (height and weight) were collected from the school-aged children using standardized procedures. Weight was measured to the nearest 0.1 kg with an electronic scale. Child height was measured to the nearest 0.1 cm using a wooden stadiometer placed on a flat surface. Regarding educational performance, 2017-year first semester scores on Amharic (local language), English, Mathematics, and Science courses as well as semester average score were extracted from the roster book of students in each school. Three supervisors and five clinical nurse data collectors were recruited for data collection. Intensive training was given for the data collectors and supervisors by the primary investigator.

### 2.4. Study Variables

Academic performance was defined as low score if less than 50%, medium score if 50%-75%, and high score if ≥75% [15]. Undernutrition was assessed according to the new WHO recommendation using stunting (height-for-age score (HAZ − score < −2SD), underweight is a weight for age (WAZ − score ≤ −2SD), and thinness (body mass index for age (BAZ) < −2SD. The four categories were defined as normal (+1 to -1SD *Z*-score), mild (between -1 and -2SD *Z*-score), moderate (between -2 and -3SD *Z*-score), and severe (less than -3SD *Z*-score) [17].

### 2.5. Data Processing and Analysis

Data were coded and entered in SPSS version 22 and exported to Stata version 14 statistical software for analysis. HAZ, WAZ, and BAZ were calculated using AnthroPlus software version 1.0.4 by the WHO 2007 growth reference standard. Descriptive statistics were used to present the findings. A bivariable analysis was done for each subject and total average score separately. Variables with *P* values of < 0.2 in the bivariable analysis were entered into a multivariable ordinal logistic regression analysis to identify the independent predictors associated with academic achievement. In the multivariable ordinal regression, the statistical significance of a variable was declared with a *P* value of < 0.05. The proportional odds assumption was checked using the Pearson chi-squared goodness-of-fit test, and data were consistent with the fitted model.

### 2.6. Ethical Consideration

The procedure of this study was approved by the Institutional Review Board of Mekelle University College of Health Sciences. Formal letters of cooperation were written to all selected public primary schools in Lalibela Town Administration. Written consent was obtained from each parent, caregivers, and directly from the school directors of all study participants. Parents and caregivers were kindly asked by formal letter to get permission before any procedure. Then, each study participant was asked for their willingness to participate in the study. Furthermore, we gave all the participants a full right to discontinue the procedures when they assumed that things were going bad with them.

## 3. Results

### 3.1. Sociodemographic Characteristics

A total of 505 school-aged children were included in the study which gives the response rate of 93%. The mean age of the participant was 9.45 ± 1.97 year. About 89.7% and 78.6% of the participants were living with their mothers and live in urban setting, respectively. Majority of them (98.6%) were Orthodox Christian. With regard to maternal characteristics, 79.3% and 46.9% of the mothers were married and illiterate on educational status, see Table 1.

### 3.2. Nutritional Status

The prevalence of stunting, underweight, and thinness was 29.5%, 35.7%, and 29.5%, respectively. Of all study participants, 89.3% ate breakfast and/or lunch before they went to school. Additionally, 55% of the study participant's household did not use iodized salt. 19.6% of the families reported that they had offered special food like fish oil for their child during childhood period.

### 3.3. School Environmental and Academic Performance

In majority, 95.4% and 88.1% of the children like their school and have a smooth relationship with teachers and school administration. Among all study participants, 74.3% study their lesson on a regular basis and 87.7% did their homework and exercise. With regard to academic achievement, among all study participants, 17.6% in Amharic, 19.2% in English, 15.8% in Science, and 26.1% in the Mathematics course had low academic achievement. Additionally, 14.7% of them had scored below 50% on the semester average, see Table 2.

### 3.4. Factors Associated with Academic Performance of School-Aged Children

In this study, five ordinal regression models were fitted; one for overall semester average and four for each course. In the multivariable analysis, place of residence, maternal educational status, family income, studying regularly, underweight, and stunting were associated with semester average achievement of school-aged children. For a child who lived in the rural, the odds of high average semester score achievement was decreased by 61% compared to those who lived in urban settings given that all of the other variables in the model are held constant (POR = 0.39; 95% CI: (0.21, 0.75**))**. Children who have mothers with secondary and above educational status had more than two times higher average semester score than children with illiterate mothers (POR = 2.12; 95% CI: (1.10, 4.07)). Additionally, a child whose family's income was from 550 to 2999 ETB had 1.71 times higher odds of achieving high average semester score (POR = 1.71; 95% CI: (1.04, 2.81)) compared to that whose income was less than 550 ETB. Compared to children who study regularly, the odds of achieving high average semester score was decreased by 51% (POR = 0.49; 95% CI: (0.29, 0.82) among students who will not do so. Concerning nutritional status, severely underweighted children have 75% decreased achievement on average semester score compared to normal weighted children (POR = 0.25; 95% CI: (0.09, 0.71)). Similarly, severely stunted children have 97% decreased average semester score compared to normal children (POR = 0.03; 95% CI: (0.01, 0.37)), see Table 3.

In the second ordinal regression model, the Science course score was modeled. Accordingly, the results reveal that achieving higher Science course score was decreased by 52% among children who lived in rural areas as compared to urban dwellers (POR = 0.48; 95% CI: (0.26, 0.90). Additionally, a child whose family's income was from 550 to 2999 ETB had 1.67 times higher odds of achieving a high Science course score (POR = 1.67; 95% CI: (1.02, 2.75)) compared to that whose income was less than 550 ETB. With regard to nutritional status, moderate underweighted children had 68% decreased odds of achieving high science score compared to normal underweighted children (POR = 0.32; 95% CI: (0.12, 0.89)). Similarly, severely stunted children had 90% decreased odds of achieving a high Science score compared to stunted children as compared to normal children (POR = 0.10; 95% CI: (0.02, 0.61)), see Table 4.

With regard to Mathematics course score, a child whose family's income was from 550 to 2999 ETB had 1.73 times higher odds of achieving a high Mathematics course score (POR = 1.73; 95% CI: (1.06, 2.81)) compared to that whose income was less than 550 ETB. Additionally, a child who did not study his/her lesson regularly had 41% decreased odds of achieving higher Mathematics course compared to their counterparts (POR = 0.59; 95% CI: (0.36, 0.97)). With regard to nutritional status moderate underweighted (POR = 0.34; 95% CI: (0.12, 0.92)) and severely stunted (POR = 0.05; 95% CI: (0.004, 0.48)) children had 64% and 95% decreased odds of scoring a high Mathematics score as compared to normal children, respectively, see Table 5.

In the multivariable analysis, ordinal logistic regression maternal education status, family size, residence, and underweight were statistically significant predictors of English and Amharic course scores. Moderate underweighted children had 66% decreased odds of achieving high English (POR = 0.34; 95% CI: (0.13, 0.91)) and Amharic (POR = 0.34; 95% CI: (0.13, 0.92)) courses score as compared to normal weighed children. Similarly, a child whose mother had secondary and above educational status has 2.02 and 2.78 times higher odds of achieving high English (POR = 2.54; 95% CI: (1.09, 3.77) and Amharic (POR = 2.78; 95% CI: (1.47, 5.26) course scores as compared to a child with an illiterate mother, respectively. A child who lives with more than five family members had 2.54 times higher odds of achieving higher English course score as compared to those who lived with less than two family members. On the other hand, a child who lived in rural areas had 65% decreased odds of achieving a high Amharic course score than those who lived in urban areas.

## 4. Discussion

This study is aimed at assessing the effect of nutritional status on academic performance of school-aged children. Accordingly, the prevalence of stunting, underweight, and thinness was 29.5%, 35.7%, and 29.5%, respectively. These study findings are lower than the findings in Sri Lanka in which 50%, 32%, and 34% of school-aged children are underweight, stunted, and thin, respectively [10]. The possible reason was the study done in Sri Lanka was done at the rural setting. The prevalence of stunting is similar with the study done in the urban settings of Fogera and Libo Kemkem districts, This need to be corrected as: However, it is different from the finding in the rural setting of Fogera and Libo Kemkem districts in which 42.7% of school-age children were stunted. The prevalence of thinness is higher than a study done at Fogera and Libo Kemkem districts, Ethiopia [18]. This difference could be due to the sociodemographic and geographic differences. The magnitude of stunting in this study is greater than the study done in a rural area of Morocco which was 22.8% [19].

Study finding revealed that a child who lives in rural area had 65%, 52%, and 61% decreased odds of high achievement in the Amharic course, Science course and average semester scores, respectively. This study finding was supported by a study done in Iran, which shows that cognitive function of children was associated with the place of residence [20]. Children who have mothers with secondary and above educational status have more than two times higher English, Amharic, and average semester scores given that all of the other variables in the model are held constant. This study finding is similar with a study done in Morocco, Malaysia, Iran, and Hawa Gelan, Southwest Ethiopia [15, 19–21]. This signifies the importance of educating females in improving the child nutritional status as well as academic achievement.

On the other hand, a child from a family with an income from 550 to 2999 ETB has 1.71, 1.67, and 1.73 times higher average semester, Science course, and Mathematics course score than that from a lower income family. The increase in household income may be linked to household food security and enhanced school attendance. This study finding was similar with a study done in Iran and Hawa Gelan, Southwest Ethiopia, where higher monthly house hold income was associated with student's academic performance [15, 20]. This may be due to family income directly influencing the nutritional status of household members. With regard to academic performance, compared to a child who study their lesson regularly, those who did not study regularly have 51% and 41% decreased odds of achieving high average semester and Mathematics course score.

With regard to nutritional status, severely underweighted children have 75% decreased odds of achieving high average semester score compared to normal weighted children. Furthermore, moderate underweighted children have 68%, 64%, 66%, and 66% decreased odds of achieving high Science, Mathematics, Amharic, and English course scores compared to normal weighted children, respectively. This study finding was similar with a study done in Nuwara Eliya Educational Zone and Hawa Gelan, Southeast Ethiopia [10, 15]. But the finding is different from a study done in Morocco that indicates WAZ did not have a significant association with academic achievement of primary school children [19]. This can be more supported by the report of Christian Perspectives in Education, while adequate and high-quality nutrition improves good academic performance, yet malnourishment contributes to low educational achievement where its prevalence is higher in developing countries [22]. This may be due to improved nutritional status being associated with better ability to learn, fewer absences, improved behavior, and causing fewer disruptions in the classroom which increases academic performance [23].

Alternatively, severely stunted children have 97%, 90%, and 95% decreased odds of achieving high average semester score, Science course score, and Mathematics course score as compared to not stunted children. This study finding is similar with a study done in Sri Lanka in which low educational performance in Tamil (Language of Sri Lanka), Mathematics, and overall subject average was significantly higher among stunted participants than normal children [10]. However, it was different from a study done in Morocco, in which stunting was positively correlated with annual score and average score of Mathematics course score [9] and Malaysia where students educational achievement was not affected by stunting [21]. This may be due to nutritional status being able to directly affect mental capacity among school-aged children [23].

This study has its own limitations. First, the students' academic performance was evaluated by semester scores and it may also vary due to pedagogical factors which were not considered in this study. Second, due to WHO AnthroPlus 2007 GS limitations, participants whose age is greater than 10 years especially females during underweight assessment were excluded due to the fact that their weight increases in spite of nutritional status but by hormonal factors during pubertal period, and as a result, the sample size became small for only underweight assessment.

## 5. Conclusion

Greater magnitude of malnutrition (stunting, underweight, and thinness) is found among primary school children in Lalibela town. This nutritional status significantly affects the educational performances of primary school children. Place of residence, family monthly income, maternal education status, and regular lesson studying, underweight, and stunting are statistically significant predictors of educational performance in Science, Amharic, English, Mathematics, and semester average scores. So, health and education sectors as well as other concerned bodies should work together to avert this adverse effect of malnutrition.

## Figures and Tables

**Table 1:** Sociodemographic and economic characteristics of the study participants in Lalibela primary schools (*N* = 505), 2017.

Variables		Frequencies	Percent (%)
Gender	Male	252	49.90
	Female	253	50.10

Is a child living with his mother?	Yes	453	89.70
	No	52	10.30

Maternal age (year)	<20	3	0.59
	20-25	44	8.71
	26-29	90	17.82
	30-35	140	27.72
	>35	228	45.15

Residence	Urban	397	78.61
	Rural	108	21.39

Maternal marital status:	Married	410	81.19
	Divorced	50	9.90
	Separated	33	6.53
	Widowed	12	2.38

Maternal religion	Orthodox	498	98.61
	Muslim	7	1.39

Family income per month (ETB)	<1000	251	49.70
	1001-2500	189	37.43
	2501 and above	65	12.87

Number of family living together	2	46	9.1
	3-5	307	60.8
	>5	152	30.1

**Table 2:** Descriptive results of academic performance of primary school children in Lalibela (*N* = 505), 2017.

Variables		Frequency	Percent
Amharic score	<50	89	17.6
	50-75	215	42.6
	>75	201	39.8

English score	<50	97	19.2
	50-75	246	48.7
	>75	162	32.1

Science score	<50	80	15.8
	50-75	220	43.6
	>75	205	40.6

Mathematics score	<50	132	26.1
	50-75	207	41.0
	>75	166	32.9

Semester average score	<50	74	14.7
	50-75	257	50.9
	>75	174	34.5

**Table 3:** Factors associated with average semester score achievement among school-aged children in Lalibela town, North Ethiopia.

Variables	Category	Semester average score achievements			Crude POR (95% CI)	Adjusted POR (95% CI)
		Poor	Medium	High		
Maternal marital status	Married	55	209	146	1	1
	Divorced	14	23	13	0.5 (0.28,0.90)^∗^	0.65 (0.30, 1.4)
	Separated	4	16	13	1.16 (0.59,2.29)	1.6 (0.64, 4.21)
	Widowed	1	9	2	0.66 (0.23,1.84)	0.96 (0.28, 3.3)

Residence	Urban	59	183	155	1	1
	Rural	15	74	19	0.54 (0.36, 0.81)^∗^	0.39 (0.21,0.75)^∗^

Maternal education status	Illiterate	38	137	62	1	1
	Primary	26	71	52	1.26 (0.85, 1.88)	1.20 (0.71, 2.03)
	Secondary and above	10	48	60	2.63(1.71, 4.05) ∗	2.12 (1.10, 4.07)^∗^

Maternal occupation	Housewife	47	177	106	1	1
	Employed	14	32	36	1.37 (0.85, 2.20)	0.90 (0.46, 1.76)
	Private business	4	22	21	1.69 (0.94, 3.03)	0.87 (0.40, 1.86)
	Other	9	26	11	0.68 (0.38, 1.23)	0.56 (0.24, 1.34)

Number of family living together	<2	15	19	12	1	1
	3-5	38	156	113	2.45 (1.31, 4.56)^∗^	1.43 (0.62, 3.29)
	>5	21	82	49	2.05 (1.07, 3.96)^∗^	1.62 (0.65, 4.05)

HH income	<550	48	137	66	1	1
	550-2999	22	95	72	1.75 (1.21, 2.52)^∗^	1.71 (1.04, 2.81)^∗^
	>3000	4	25	36	3.5 (2.04, 6.03)^∗^	1.32 (0.61, 2.85)

HH iodized salt use	Yes	33	97	98	1	1
	No	41	160	76	0.61 (0.43, 0.85)^∗^	0.87 (0.54, 1.41)

Special foods took during childhood	Yes	9	39	51	1	1
	No	65	218	123	0.43 (0.28, 0.66)∗	0.74 (0.41, 1.33)

Regular study	Yes	48	191	136	1	1
	No	26	66	38	0.67(0.46,0.99) ∗	*0.49(0.29,0.82)*∗

Under weight	Normal	3	64	35	1	1
	Mild	8	59	54	1.29(0.78,2.11)	0.90(0.46,1.77)
	Moderate	27	39	24	0.39(0.22,0.68) ∗	*0.25(0.09,0.71)*∗
	Severe	11	12	11	0.43(0.19,0.95) ∗	0.61(0.15,2.47)

Stunting	Normal	11	108	56	1	1
	Mild	17	96	69	1.12 (0.76, 1.65)	1.48 (0.84, 2.61)
	Moderate	32	46	41	0.64 (0.40, 1.01)	0.89 (0.37, 2.15)
	Severe	14	7	8	0.24 (0.11, 0.56)	0.03 (0.01, 0.37)

Thinness	Normal	14	115	72	1	1
	Mild	24	75	56	0.83 (0.56, 1.24)	1.22 (0.64, 2.32)
	Moderate	23	45	36	0.67 (0.42, 1.06)	1.49 (0.62, 3. 60)
	Severe	13	22	10	0.38 (0.20, 0.71)	0.64 (0.20, 2.02)

**Table 4:** Factors associated with Science course achievement among school-aged children in Lalibela town, North Ethiopia.

Variables	Category	Science course achievements			Crude POR (95% CI)	Adjusted POR (95% CI)
		Poor	Medium	High		
Maternal marital status	Married	57	184	169	1	1
	Divorced	16	16	18	0.55 (0.31, 0.99)	0.85 (0.39, 1.87)
	Separated	4	14	15	1.18 (0.60, 2.31)	1.77 (0.70, 4.52)
	Widowed	3	6	3	0.49 (0.17, 1.43)	0.67 (0.19, 2.35)

Residence	Urban	62	155	180	1	1
	Rural	18	65	25	0.52 (0.35, 0.77)	0.48 (0.26, 0.90)^∗^

Maternal education status	Illiterate	41	114	82	1	1
	Primary	26	63	60	1.18 (0.80, 1.73)	1.23 (0.73, 2.08)
	Secondary and above	13	43	62	1.98 (1.29, 3.03)	1.34 (0.71, 2.54)

Maternal occupation	Housewife	48	158	124	1	1
	Employed	15	26	41	1.36 (0.85, 2.18)	1.09 (0.55, 2.15)
	Private business	7	16	24	1.51 (0.83, 2.73)	0.91 (0.42, 1.97)
	Other	10	20	16	0.79 (0.44, 1.41)	0.69 (0.29, 1.65)

Number of family living together	< 2	12	20	14	1	1
	3-5	46	131	130	1.83 (1.01, 3.29)	1.04 (0.47, 2.30)
	>5	22	69	61	1.73 (0.92, 3.30)	1.74 (0.73, 4.15)

HH income	<550	47	124	80	1	1
	550-2999	27	76	86	1.65 (1.15, 2.37)^∗^	1.67 (1.02, 2.75)^∗^
	>3000	6	20	39	2.98 (1.73, 5.13)^∗^	1.29 (0.59, 2.83)

HH iodized salt use	Yes	36	81	111	1	1
	No	44	139	94	0.64 (0.46, 0.89)	0.71 (0.44, 1.14)

Does the child take special foods?	Yes	11	34	54	1	1
	No	69	186	151	0.51 (0.33, 0.79)	0.92 (0.51, 1.66)

Sick with diarrhea	Yes	22	60	42	1	1
	No	58	160	163	1.37 (0.94, 2.00)	1.40 (0.84, 2.33)

Regular study	Yes	54	164	157	1	1
	No	26	56	48	0.76 (0.52, 1.12)	0.65 (0.39, 1.08)
	No	42	69	52	0.49 (0.34, 0.71)^∗^	0.70 (0.41, 1.20)

Does the child like the school environment?	Yes	74	207	201	1	1
	No	6	12	4	0.40 (0.18, 0.88)	0.46 (0.17, 1.21)

Under weight	Normal	6	55	41	1	1
	Mild	8	52	61	1.37 (0.83, 2.25)	0.85 (0.44, 1.66)
	Moderate	24	35	31	0.51 (0.30, 0.89)	0.32 (0.12, 0.89)^∗^
	Severe	9	16	9	0.41 (0.19, 0.86)	0.59 (0.15, 2.33)

Stunting	Normal	15	89	71	1	1
	Mild	15	81	86	1.23 (0.84, 1.82)	1.68 (0.95, 2.98)
	Moderate	36	44	39	0.48 (0.30, 0.75)	0.64 (0.27, 1.53)
	Severe	14	6	9	0.25 (0.11, 0.57)	0.10 (0.02, 0.61)^∗^

Thinness	Normal	21	101	79	1	1
	Mild	25	58	72	1.11 (0.75, 1.65)	1.75 (0.92, 3.31)
	Moderate	23	37	44	0.85 (0.54, 1.34)	1.95 (0.83, 4.58)
	Severe	11	24	10	0.46 (0.25, 0.83)	0.60 (0.38, 1.89)

**Table 5:** Factors associated with Mathematics course achievement among school-aged children in Lalibela town, North Ethiopia.

Variables	Category	Mathematics course achievements			Crude POR (95% CI)	Adjusted POR (95% CI)
		Poor	Medium	High		
Maternal marital status	Married	103	168	139	1	1
	Divorced	17	19	14	0.70 (0.40, 1.21)	0.91 (0.43, 1.93)
	Separated	7	14	12	1.16 (0.60, 2.23)	1.28 (0.52, 3.12)
	Widowed	5	6	1	0.38 (0.13, 1.07)	0.41 (0.12, 1.43)

Maternal education status	Illiterate	68	103	66	1	1
	Primary	45	58	46	1.04 (0.71, 1.52)	0.80 (0.48, 1.34)
	Secondary and above	19	45	54	2.12 (1.10, 3.2)	1.85 (0.98, 3.49)

Maternal occupation	Housewife	85	146	99	1	1
	Employed	25	22	35	1.26 (0.79, 2.00)	0.88 (0.45, 1.72)
	Private business	7	21	19	1.65 (0.94, 2.88)	0.88 (0.42, 1.85)
	Other	15	18	13	0.81 (0.46, 1.44)	0.77 (0.33, 1.79)

Number of family living together	<2	18	18	10	1	1
	3-5	73	124	110	2.05 (1.15, 3.67)	1.60 (0.72, 3.53)
	>5	41	65	46	1.66 (0.90, 3.06)	1.94 (0.81, 4.64)

Residence	Urban	104	145	148	1	1
	Rural	28	62	18	0.62 (0.42, 0.91)	0.59 (0.32, 1.07)

HH income	<550	75	114	62	1	1
	550-2999	48	68	73	1.58 (1.11, 2.25)	1.73 (1.06, 2.81)
	>3000	9	25	31	2.60 (1.56, 4.36)	1.39 (0.65, 2.95)

HH iodized salt use	Yes	54	82	92	1	1
	No	78	125	74	0.64 (0.46, 0.88)	0.96 (0.60, 1.53)

Deworming	Yes	99	166	135	1	1
	No	33	41	31	0.77 (0.52, 1.15)	0.61 (0.38, 1.04)

Does the child eat breakfast/lunch before school?	Yes	113	187	151	1	1
	No	19	20	15	0.68 (0.40, 1.15)	1.29 (0.60, 2.77)

Does the child take special foods?	Yes	16	38	45	1	1
	No	116	169	121	0.50 (0.33, 0.76)	0.85 (0.48, 1.49)

Regular study	Yes	86	156	133	1	1
	No	46	51	33	0.58 (0.40, 0.84)	0.59 (0.36, 0.97)

Does the child like the school environment?	Yes	121	201	160	1	1
	No	10	6	6	0.51 (0.22, 1.16)	0.99 (0.36, 2.73)

Under weight	Normal	9	60	33	1	1
	Mild	16	54	51	1.20 (0.74, 1.93)	0.93 (0.49, 1.77)
	Moderate	40	21	29	0.41 (0.24, 0.71)	0.34 (0.12, 0.92)^∗^
	Severe	15	9	10	0.39 (0.18, 0.83)	0.66 (0.17, 2.58)

Stunting	Normal	21	102	52	1	1
	Mild	39	73	70	1.03 (0.71, 1.51)	1.58 (0.91, 2.75)
	Moderate	55	26	38	0.45 (0.29, 0.70)	0.63 (0.27, 1.49)
	Severe	17	6	6	0.23 (0.10 ,0.51)	0.05 (0.004, 0.48)^∗^

Thinness	Normal	34	102	65	1	1
	Mild	44	55	56	0.86 (0.58, 1.26)	1.33 (0.71, 2.47)
	Moderate	36	34	34	0.67 (0.43, 1.05)	1.36 (0.58, 3.18)
	Severe	18	16	11	0.48 (0.26, 0.87)	0.99 (0.33, 3.04)

## Data Availability

We confirmed that all data underlying the findings would be fully available without restriction if the manuscript is published.
