# Corrigendum to “Nutritional Status and Educational Performance of School-Aged Children in Lalibela Town Primary Schools, Northern Ethiopia”

**DOI:** 10.1155/2020/1826079

**Published:** 2020-10-12

**Authors:** Muluken Ayalew, Alemayehu Bayray, Abate Bekele, Simegnew Handebo

**Affiliations:** ^1^Lalibela Hospital, Lalibela, Ethiopia; ^2^School of Public Health, College of Health Sciences, Mekelle University, Mekelle, Ethiopia; ^3^Department of Health Education and Behavioral Sciences, Institute of Public Health, College of Medicine and Health Sciences, University of Gondar, Gondar, Ethiopia

In the article titled “Nutritional Status and Educational Performance of School-Aged Children in Lalibela Town Primary Schools, Northern Ethiopia” [1], author Simegnew Handebo was affiliated to the Department of Health Education and Behavioral Sciences, College of Medicine and Health Sciences, Institute of Public Health, Gondar University, Gondar, Ethiopia, which is incorrect. The correct affiliation for this author is Department of Health Education and Behavioral Sciences, Institute of Public Health, College of Medicine and Health Sciences, University of Gondar, Gondar, Ethiopia.
